# Genetic algorithm for parameter optimization of supercapacitor model

**DOI:** 10.1371/journal.pone.0325645

**Published:** 2025-07-17

**Authors:** Filipe Menezes, Sérgio Cunha, William Assis, Allan Manito, Reinaldo Leite, Thiago Soares, Hugo Lott

**Affiliations:** 1 Electrical and Biomedical Engineering Faculty, Institute of Thecnology, Federal University of Pará, Belém Pennsylvania, Brazil; 2 Norte Energia, Pará, Brazil; University of Jammu, INDIA

## Abstract

Electric energy storage systems have advanced significantly in recent years, driven by the growing expansion of renewable energy sources, the rise of electromobility, and other emerging configurations within the current electrical energy system. Among the various energy storage technologies, supercapacitors have gained considerable attention. Due to their ability to deliver large amounts of power over short periods, supercapacitors can be highly effective in hybrid storage systems, for example, enhancing overall system performance. Therefore, detailed studies on supercapacitors and their electrical circuit models have been developed with the aim of representing them as close as possible to actual physical behavior for numerous applications, such as in the context of Digital Twin (DT), an application that will support the monitoring of the operation and health of the supercapacitor throughout its useful life. The present work aims to estimate optimally some parameters of an electrical circuit model of a supercapacitor, in such a way as to obtain responses with very low errors and, thus, be able to use this computational electrical modeling for the development of a Digital Twin system. For the optimal adjustment of the electrical circuit model parameters, a Genetic Algorithm (GA) is used. The response of the electrical circuit, adjusted by the Genetic Algorithm (GA), is then compared to the response obtained through computer simulation of a supercapacitor using PSIM software, which is a software well validated in such studies. The results demonstrated strong alignment between the response using GA and the response using PSIM. Specifically, the charge and discharge curves of the supercapacitor, obtained through GA adjustment and PSIM simulation, were very similar, showing an error of just 2.2%. Thus, the supercapacitor model adjusted via GA demonstrates a good response to the physical phenomenon in question and can be used to develop a Digital Twin (DT) system, aiding in the operational and health monitoring of the supercapacitor.

## 1. Introduction

Supercapacitors are devices that operate based on the amount of energy stored as ions between the electrolyte and electrode. We can divide supercapacitors in three types based on its energy storage mechanism, namely: electrochemical double-layer capacitor, pseudo-capacitor and hybrid supercapacitor [1].The supercapacitor that accumulates electric charges at the interface between electrode pores and the electrolyte is the electrochemical double-layer supercapacitor or electric double layer supercapacitor (EDLC). This phenomenon occurs at the interface between the pores of the electrodes and the electrolyte, which provides a larger specific area to the electrodes. As a result, supercapacitors achieve high capacitance compared to their electrolytic counterparts [2]. They are composed of: (i) a pair of active material electrodes adhered to a current collector with terminal connections for the electronic circuit, (ii) a membrane that physically and electrically isolates both electrodes, (iii) electrolytes containing charged ions generally soaking the separator membrane, and (iv) the encapsulation [3]. Supercapacitors respond more quickly to charge variations compared to lithium-ion batteries due to their high power density characteristics, and have attracted much attention in SC technology [4–9].

In recent years, energy storage systems have experienced significant growth, both in quantity and technological advancements, playing an increasingly prominent role in electrical energy systems. One particularly promising technology is the use of supercapacitors for electrical energy storage, offering advantages such as high power delivery in extremely short periods, excellent reversibility, fast charge and discharge capabilities, and long lifespan [9]. Consequently, supercapacitors have been widely applied in power systems, including wind farms for output power stabilization, dynamic voltage regulators, distributed generation systems, and large-scale storage solutions [10,11].

Recent studies have focused on developing more accurate and efficient models for supercapacitors, aiming to optimize their performance across various applications.

In the work developed by [12], two supercapacitor models were implemented, one model using Simulink 7.5 software and the other one using Orcad 9.2 software. The parameters of the models were obtained through two measurement tests on a real supercapacitor. Comparing the models, the authors concluded that both models presented good results, and that the parameter extraction method was accurate.

In [13], two different charge/discharge sequences were proposed to extract parameters from a supercapacitor model, considering a faster and a slower process of charge/discharge. With this, the authors were able to estimate different values of tuning capacitance (Cfit), for fast charge/discharge and slow charge/discharge. Another parameter estimated in this work is the tuning resistance (Rfit), which is estimated empirically in the PSpice software, comparing the curve obtained via simulation and via experimental testing. This work was an advance compared to the work developed by [14], in which the parameters of the supercapacitor model are identified considering only one charge and discharge sequence.

The work presented in [15] introduces an innovative algorithm for extracting optimal parameters, emphasizing the role of optimization techniques in enhancing the accuracy of supercapacitor models. Additionally, [15] explores experimental methods for parameter estimation, reinforcing the necessity of experimental validation to ensure the effectiveness of theoretical models. The development of robust models for real-world applications requires a seamless integration of both experimental and theoretical approaches.

Another significant advancement is presented in [16], which leverages machine learning algorithms such as Support Vector Machine (SVM) and Particle Swarm Optimization (PSO) to model supercapacitor behavior. This hybrid approach combines artificial intelligence techniques with traditional modeling methods, resulting in more precise models that adapt to varying operational conditions.

In [17], a genetic algorithm (GA) was used to optimize some physical parameters of a supercapacitor, including the selection of electrode materials, the design of electrode structure, the composition and concentration of electrolyte, and the control of operating temperature. The main goal is to maximize the energy density and cycle life of the supercapacitor by using genetic algorithm to optimize the supercapacitor parameters design.

These studies illustrate the continuous evolution of supercapacitor modeling and characterization, underscoring the importance of advanced optimization techniques and experimental validation. The application of these methodologies can lead to substantial improvements in the performance and efficiency of energy storage systems, reinforcing the viability of supercapacitors for industrial applications and renewable energy generation.

The implementation of Digital Twins [18] in energy storage systems involving supercapacitors holds tremendous potential for enhancing accuracy and efficiency. Digital Twins are virtual representations of physical systems that enable real-time simulation, monitoring, and optimization. In the context of supercapacitors, Digital Twins facilitate modeling and predicting device behavior under diverse operating conditions, allowing for parameter identification and optimization.

The use of advanced optimization algorithms, as discussed in [15–17], is crucial for the development of accurate and effective Digital Twins. These algorithms enable precise parameter identification for supercapacitor models, which is fundamental for simulating and predicting device performance. The integration of machine learning techniques such as SVM and PSO further enhances the adaptability of Digital Twins to operational variations, improving prediction accuracy.

Moreover, the experimental approach detailed in [12–14] plays a key role in validating theoretical models, ensuring that Digital Twins accurately represent real-world supercapacitor behavior. The combination of experimental and theoretical methodologies fosters the development of robust and reliable models, which are essential for the successful deployment of Digital Twins.

However, despite the advancements achieved with SVM and PSO, certain limitations persist. While these methods are promising, they may struggle with flexibility and adaptability to new operating conditions. In contrast, Genetic Algorithms (GAs) [17] present significant advantages in this context. GAs are particularly effective in exploring complex and nonlinear solution spaces—intrinsic characteristics of supercapacitor models. Furthermore, GAs promote greater solution diversity through genetic mutations and crossovers, helping to prevent premature convergence to local optima and enhancing model robustness.

Thus, research on parameter optimization for supercapacitor models using Genetic Algorithms and other advanced optimization techniques is of great importance for the effective implementation of Digital Twins. Addressing the gaps identified in the literature and developing more precise and efficient parameter identification methods will contribute significantly to the advancement of this technology, ultimately improving the performance and efficiency of energy storage systems.

## 2. Methods

### 2.1. The proposed methodology

This manuscript presents a methodology for estimating the parameters of a supercapacitor’s electrical model using a genetic algorithm (GA). The proposed approach consists of the following steps:

1. **Supercapacitor Circuit Modeling:** The equivalent circuit of the supercapacitor is modeled using PSIM software. It is important to note that PSIM is a validated software and will serve as the reference for this study, ensuring the reliability of the results obtained through the proposed GA method. Due to technical constraints preventing the construction of an experimental setup, a computer simulation in PSIM is conducted to validate the GA-based estimation of the **Rfit** and **Cfit** parameters, as shown in Fig. 2.
2. **Simulation of the Operating Cycle:** Once the SC equivalent circuit is modeled, a simulation is performed to capture its complete operating cycle, including charging, self-discharge, and discharging phases.
3. **Parameter Calculation from Simulation Data:** After obtaining the supercapacitor’s charging and discharging curves, two key parameters of the electrical model—capacitance (**Csc**) and equivalent series resistance (**Resr**)—are calculated. Up to this stage, the results derived from the simulation could also be obtained experimentally, as they would in a real-world scenario.
4. **Optimization Using Genetic Algorithm:** In this step, the GA is employed to determine optimal values for **Rfit** and **Cfit**. The results obtained via GA are compared with the reference curve generated in PSIM, which acts as a substitute for actual experimental measurements.
5. **Fine-Tuning of Self-Discharge Resistance:** A fine adjustment is then performed to determine an appropriate value for **Rsd**. This involves modifying the resistance value and comparing it with the reference self-discharge curve obtained from the PSIM simulation.
6. **Final Validation and Comparison:** Lastly, a comparison is made between the reference operating cycle obtained in PSIM and the cycle generated using the parameters (**Rfit** and **Cfit**) optimized by the GA. This optimized model is also simulated in PSIM. A small error margin indicates that the computational model of the supercapacitor is suitable for future applications, such as digital twin implementations.

**Fig 1:**
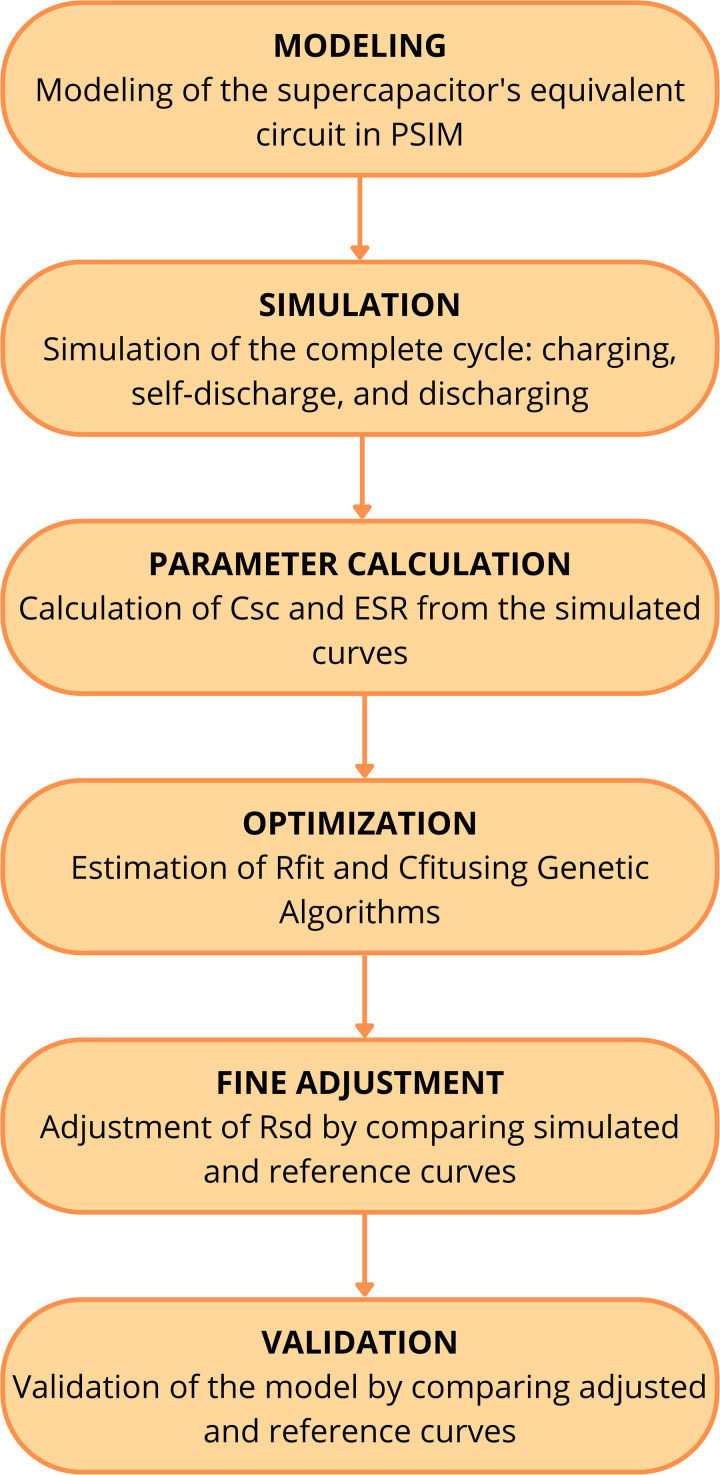
Flowchart of the proposed methodology.

Fig 1 presents a flowchart illustrating the proposed methodology.

### 2.2. Equivalent circuit in PSIM software

**Fig 2:**
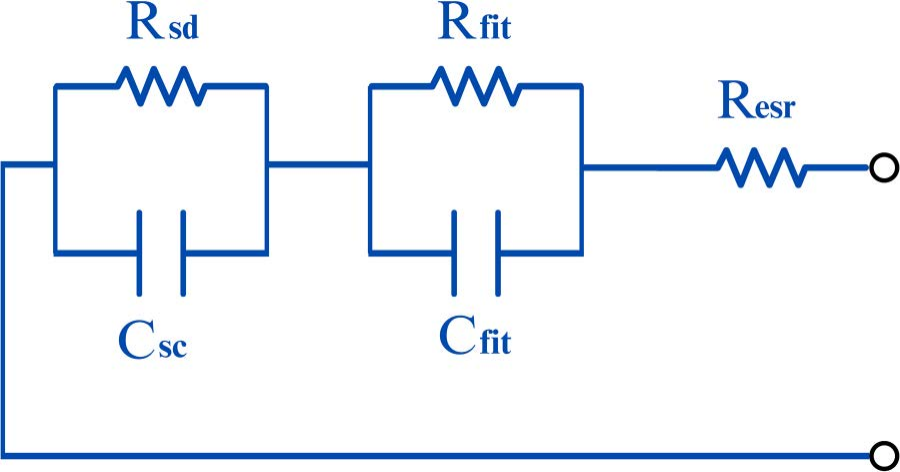
Equivalent electrical model of SC. Source: adapted from [13].

A supercapacitor (SC) can be modeled based on the equivalent circuit proposed in [13], as illustrated in Fig 2.

The equivalent series resistance (**Resr**) and capacitance (**Csc**) are determined from the supercapacitor’s operating cycle, which includes charging, self-discharge, and discharging phases. These parameters are estimated under the assumption of a constant current during the charging and discharging stages. Once the voltage curve is obtained from the SC’s operating cycle, these two parameters can be computed using specific equations, which will be presented later.

The resistance (**Rfit**) and capacitance (**Cfit**) can be determined using different methods, such as the least squares method. In this study, a genetic algorithm (GA) was adopted to estimate these parameters, iteratively adjusting them until an optimal solution is found. The application of GA is detailed in Section 2.4.

After determining the aforementioned parameters, the resistance (**Rsd**) is fine-tuned by making small adjustments and comparing the resulting self-discharge curve with the reference curve obtained from the simulated SC operating cycle.

The parameters are defined as follows:

- **Resr**: Equivalent series resistance.
- **Csc**: Capacitance associated with stored energy.
- **Cfit** and **Rfit**: Parameters used to adjust the SC’s dynamic response during charging and discharging.
- **Rsd**: Resistance associated with the SC’s self-discharge process.

The literature presents various electrical models for supercapacitors, each tailored to specific applications, reflecting the complexity of simulating their nonlinear behavior. In this work, we adopt Ciocan’s model [13], as depicted in Fig 2.

The parameter values for the computational model shown in Fig 2 were obtained from [15] and are listed in Table 1.

**Table 1:** Key parameters of the supercapacitor from [19].

C_SC_	R_ESR_	C_fit_	R_fit_	R_sd_
32 F	0.02 Ω	121.56 F	0.3184 Ω	400 Ω

Where:

- **Resr** represents the equivalent series resistance.
- **Csc** denotes the capacitance associated with stored energy.
- **Cfit** and **Rfit** are linked to adjusting the dynamic response of the SC during charging and discharging.

**Rsd** is related to the SC’ self-discharge process

The values of the computational model parameters presented in Fig 2 were taken from [19] and are presented in Table 1.

The supercapacitor used in the simulations consist in a series association of two 62 F/125 V modules, manufactured by Nesscap, with electrical parameters summarized in Table 2. This is the same model and configuration used by [19]. Each module consists of 48 cylindrical SCs of 2.7 V and 3000 F. The estimated useful life of these cells is 1,000,000 cycles at room temperature. Their construction is based on activated carbon electrodes and uses an organic electrolyte.

**Table 2:** Specifications of the used supercapacitor (EDLC).

Specifications	Module	Capacitor Bank
Capacitance	62 F	31 F
Operating Voltage	125 V	250 V
Surge Voltage	136,8 V	273,6 V
Equivalent Series Resistance (ESR)	< 15 mΩ	< 30 mΩ
Energy Density	2,36 Wh/kg	4,72 Wh/kg

With the supercapacitor modeled in PSIM, a simulation was performed to obtain its complete operating cycle, including the charging, self-discharge, and discharging phases. It is important to note that both the charging and discharging processes were conducted under constant current conditions to accurately determine the equivalent series resistance (**Resr**) and capacitance (**Csc**).

### 2.3. Simultion of the operating cycle

The simulation of the operating cycle was conducted under constant current charge-discharge (CCCD) conditions, where the supercapacitor was charged with a 10 A constant current until its voltage reached 200 V. Following the charging phase, the supercapacitor remained in self-discharge mode for 10 minutes before being discharged at a constant current. This process allows for the evaluation of the device’s performance, charge storage capacity (**Csc**) over time, and equivalent series resistance (**Resr**) [3].

**Fig 3:**
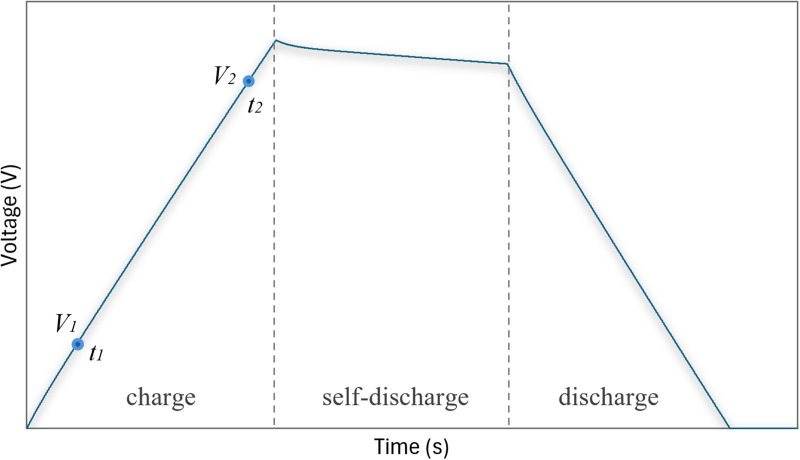
Typical CCCD graph for SCs with steady-state voltage drop: Complete cycle of charge, self-discharge, and discharge.

Fig 3 illustrates the three stages of the test: charging, self-discharge, and discharging. During the charging phase, the supercapacitor’s voltage gradually increases, as shown in Fig 3. At this stage, two voltage points (**V1** and **V2**) are recorded at 20% and 90% of the full charge, respectively, which will be used for the subsequent determination of the **Csc** parameter.

During the self-discharge phase, a gradual loss of charge occurs over time, even without an external load. This phenomenon, influenced by material quality and internal resistance, directly affects efficiency. Excessive self-discharge can significantly limit the application of supercapacitors in energy storage systems.

**Fig 4:**
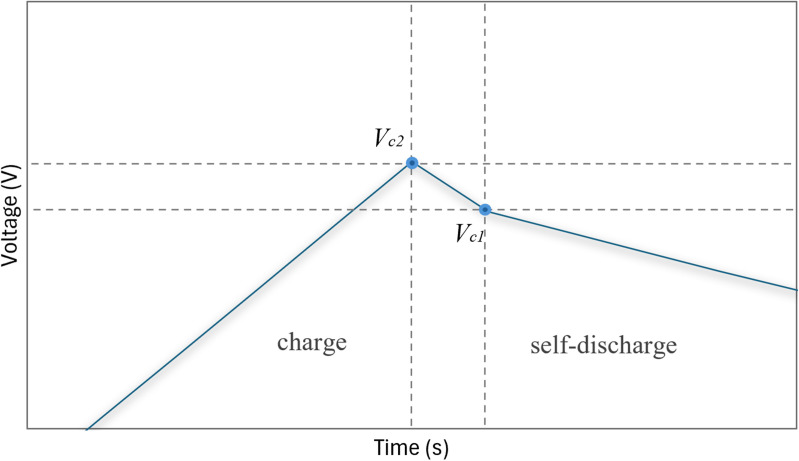
Typical CCCD graph for SCs with steady-state voltage drop: slight voltage drop immediately after charging ended. The data obtained from the charge and discharge curve are crucial for determining the capacitance (Csc) and equivalent series resistance (Resr). The data extracted from Figs 3 and 4 are applied to the following Eqs (1) and (2).


In the discharge phase, the voltage decreases as stored energy is released, and the discharge curve provides valuable insights into charge storage capacity and internal resistance (**Resr**). Fig 4 highlights the slight voltage drop caused by internal resistance immediately after the charging phase. The difference between **VC2** and **VC1** will be used to calculate **Resr**.

$$C_{sc}=\frac{I_{c}\Delta t}{\Delta V}=\frac{I_{c}(t_{2}-t_{1})}{\left(V_{2}-V_{1}\right)}$$ (1)

$$R_{esr}=\frac{\Delta V}{\Delta I}=\frac{V_{c2}-V_{c1}}{I_{c}}$$ (2)

Which Ic is the charging current.

It is important to mention that in a real practical approach, the supercapacitor voltage curve shown in Fig 3 is obtained experimentally. In the present work, due to the lack of an experimental bench, this voltage curve was obtained by computer simulation in PSIM software.

Furthermore, the data from the charging phase will serve as the foundation for calculating the genetic algorithm’s fitness. This calculation will aid in determining the most suitable dynamic parameters (Cfit and Rfit) for the supercapacitor model.

### 2.4. Genetic algorithm optimization

The use of a genetic algorithm (GA) was used to adjust the Rfit and Cfit values with the objective of obtaining a minimum error between the response through PSIM simulation (reference voltage curve with pre-defined Rfit and Cfit) and the response with Rfit and Cfit values obtained via GA, which this last one will be determined using the supercapacitor charging equation, shown later in this work.

**Fig 5:**
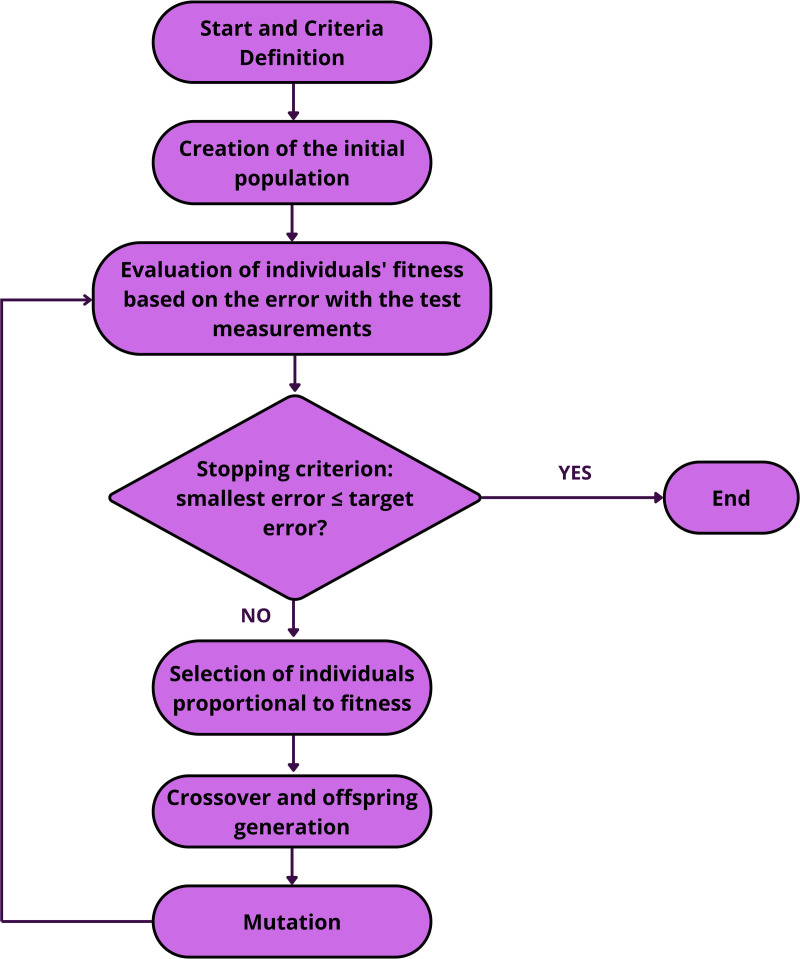
Genetic Algorithm Flowchart.

Based on [20], a genetic algorithm was developed, and its flowchart is presented in Fig 5, in which each step is described in the following subtopics.

#### 2.4.1. AG criteria definition.

In the first stage of GA, the fundamental parameters are defined and the calculations necessary for the correct execution of the program are carried out. Initially, the number of individuals per generation is specified, representing the number of possible solutions analyzed in each generation of the GA. To guarantee genetic variability, a total of 1000 individuals per generation was adopted, while the total number of generations was defined as 100 thousand, implying that if the stopping criterion, to be discussed later, is not reached, the program is terminated at limit generation. Furthermore, the variables (chromosomes) that the GA will optimize are established: Rfit and Cfit, totaling two variables.

Furthermore, a range of search values is defined for these parameters, delimiting the search space between 0 and 100 thousand. This approach contributes to algorithm efficiency by avoiding the creation of physically improbable solutions for resistance and capacitance, which reduces computational effort.

Another relevant parameter is the precision adopted for the binary coding of values, allowing continuous intervals to be represented with a specificity of 5 decimal places.

Additionally, the previously calculated parameters (Resr and Csc) are declared to compose the GA fitness function.

Therefore, the code converts the defined values range size to binary base, considering the desired precision. This results in approximately 34 bits to represent each variable, totaling 68 bits for the two variables to be optimized. Table 3 summarizes the initial parameters used in the proposed GA.

**Table 3:** Initial GA Criteria.

Criterion	Value
Maximum generations number	100,000
Chromosomes	2
Solution values ranger	[0, 100,000]
Decimal precision	5
Bits (Genes)	68
Csc	Defined by Eq (1)
Resr	Defined by Eq (2)
Charging current	Obtained in the CCCD test
Charging voltage curve	Obtained in the CCCD test

#### 2.4.2. Initial population.

In this algorithm stage, the GA creates the first generation of individuals that will be analyzed and evolved throughout the process. Each individual represents a possible problem solution, and its characteristics are encoded in a 68-bit binary array, as defined in the previous step. The total number of individuals in this initial population is defined in the initial criteria, which is 1,000.

To generate each individual, each bit (gene) is determined by the algorithm based on a random value uniformly distributed between 0 and 1. If the value is less than 0.5, the corresponding bit is set to 0, otherwise it is set as 1. This process ensures that the initial population is generated randomly, distributing individuals in the search space.

**Fig 6:**
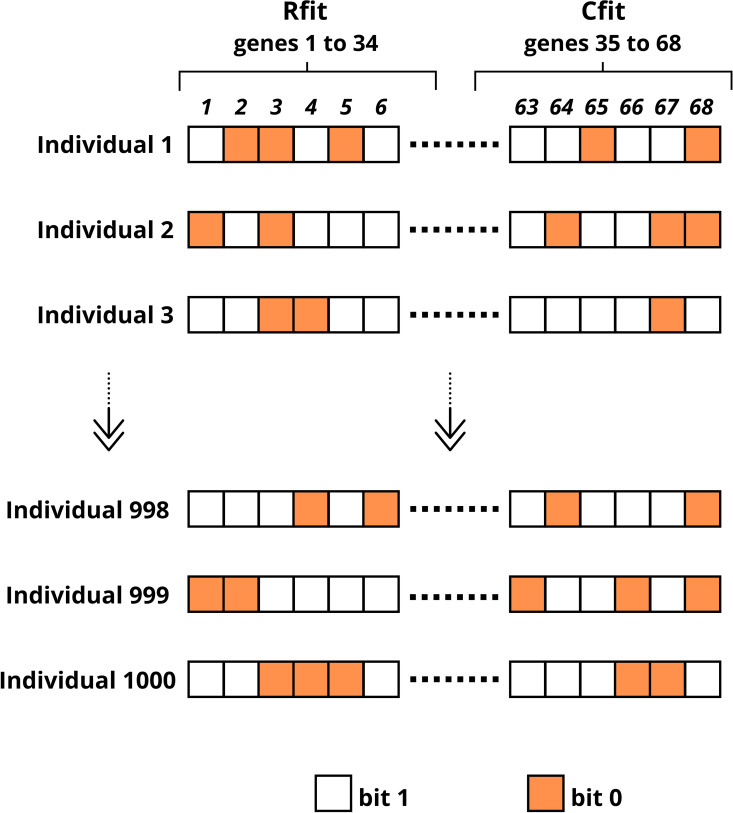
Initial population example.

In Fig 6, the proposed GA initial population is presented.

#### 2.4.3. Fitness function.

In this step, the algorithm analyzes each individual in the initial population to determine its fitness based on minimizing the error between the simulation with the parameters estimated via GA and the simulation with the reference data. To do this, the binary values of each individual are converted to their real equivalents, representing the resistance (Rfit) and capacitance (Cfit) values.

Then, the fitness of each individual is calculated, which indicates the quality of each solution to the problem. This fitness is determined by the inverse of the average error between the voltage data collected during the charging stage in the CCCD test (reference simulation via PSIM – Vsc target) and the charging voltage calculated in GA (Eq (3) – Vsc modeled), the latter being calculated for each individual.


$$V_{SC}(t)=u\left[\frac{t}{b_{1}}-\frac{b_{2}-a_{1}b_{1}}{b_{1}^{2}}+\left(\frac{b_{2}^{2}-a_{1}b_{2}b_{1}+a_{2}b_{1}^{2}}{b_{2}b_{1}^{2}}\right)e^{-\frac{b_{1}}{b_{2}}t}\right]$$ (3)

Where:

u=amplitude of the applied current step sc during the charging stage

$a_{1}=R_{ESR}C_{SC\;}+R_{fit}C_{fit}+R_{fit}C_{SC\;}$

$a_{2}=R_{ESR}C_{SC}R_{fit}C_{fit}$

$b_{1}=C_{SC\;}$

$b_{2}=C_{SC\;}R_{fit}C_{fit}$

The fitness equation for of an individual α is defined by Eq (4):


$$Z(\alpha )=\frac{1}{Avarage\;Error(\alpha )+\varepsilon }$$ (4)

Where ε is a very small number (2 ⁻ ⁵²) included to prevent division by zero in case the mean error is extremely close to zero. The average error is given by Eq (5):


$$Avarage\;Error=\;\frac{1}{t}\sum_{i=1}^{t}\left|V_{sc\_target}-V_{sc\_modeled}\right|\mathrm{\backslash ]}$$ (5)

Based on average error, the GA analyzes the stopping criterion. At each generation, the best individual average error is compared with a previously defined target error, which represents an acceptable limit for the difference between the estimated data and the reference data. If the best average error is less than or equal to the target error, the algorithm terminates the main loop and displays the optimized values. This approach optimizes the algorithm execution, avoiding unnecessary processing of additional generations when the desired solution is found. However, if the stopping criterion is not achieved, the algorithm saves the individual with the lowest error for the next generation and only exchanges it if a better individual emerges in the next generations.

Regarding the target error, used as a stopping criterion, the value of 0.5% was adopted. This value was chosen based on numerous and extensive algorithm executions, in which it was observed to be an adequate value to extract a very small error from the routine, ensuring that the SC model comes as close as possible to the target behavior.

#### 2.4.4. Selection.

The algorithm selects the fittest individuals from the population and forms pairs for crossing, using a criterion proportional to fitness, that is, the greater the fitness, the greater the chance of the individual being selected.

First, the fitness of each individual, Z(α), is normalized in relation to the total sum of fitness population, converting it into a percentage, Z%(α), as shown in Eq (6).


$$Z_{\;\%}(\alpha )=\frac{Z(\alpha )}{\sum_{\alpha =1}^{ind}Z(\alpha )}\times 100$$ (6)

Where *ind* is the number of individuals in the population.

This process ensures that each individual receives a relative probability of being selected, proportional to their fitness. The next step consists of constructing a cumulative vector, which stores the accumulated probabilities. For each individual, the accumulated value is obtained by adding its normalized fitness with the accumulated sum of previous individuals.

**Fig 7:**
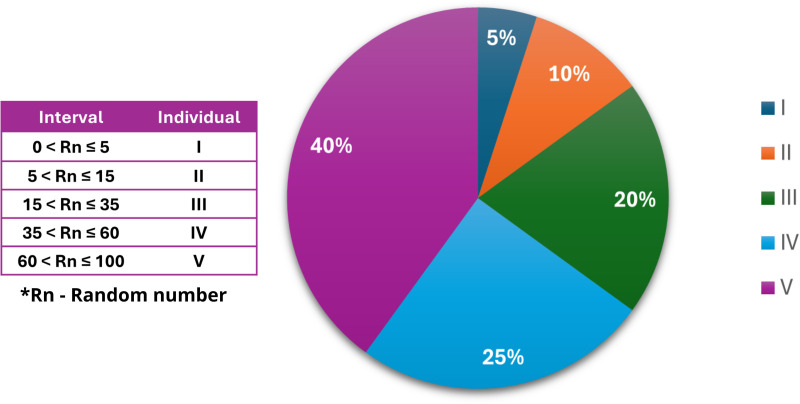
Example of accumulated skills distribution per individual and individual selection probability.

To form a pair, two random numbers are used to select individuals from the population. These numbers are compared to the accumulated vector of probabilities and the corresponding individual is the one whose accumulated range contains the generated number. For example, if the accumulated vector is [5,14, 35, 60, 100] and the generated number is 25, the third individual will be chosen, as the number 25 is in the range between 15 and 35. Fig 7 illustrates the distribution of these intervals and their weight for the selection. This process is known as the roulette method and ensures that individuals with greater fitness are more likely to be selected, but without completely excluding those who are less apt.

This procedure is repeated twice, once for each individual in the pair, ensuring that both members are chosen proportionally to their fitness.

#### 2.4.5. Crossing.

For crossing, the pairs of individuals selected in the previous phase are combined to generate new individuals, promoting the combination of genetic characteristics between them. The crossing is carried out using a cutoff point, which determines the position in the binary code where information from the two individuals will be exchanged. For each pair formed, the cutoff point is defined randomly and divides the genetic code into two parts: the genes located before the cutoff point are kept from the first individual, while the genes located after the cutoff point are exchanged with those of the second individual.

**Fig 8:**
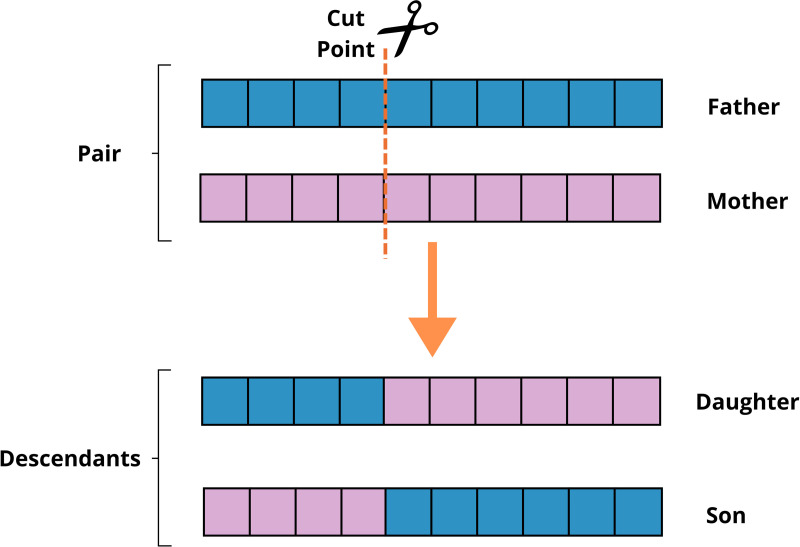
Crossing mechanism.

Fig 8 illustrates the process, which works as follows:

For genes located after the cutoff, the father’s binary data (genes) are transferred to the daughter, while the mother’s data is transferred to the son.

For genes located before the cutoff point, the opposite occurs: the mother’s binary data is transferred to the daughter, and the father’s data is transferred to the son.

This exchange of genetic information results in two new individuals (descendants), who inherit characteristics from both parents. The crossover process leads to a problem solution convergence, however, the solution may get stuck in a local optimal solution. To overcome this situation, mutation is performed on individuals, with the aim of achieving a global optimal solution.

The crossover rate used in the present work was 0.8, that is, once the pair is selected in the selection stage, the probability of crossover is 80%.

At the end of the crossing stage, the population is made up of the descendants generated, which proceed to the next steps of the algorithm, such as mutation and fitness evaluation.

#### 2.4.6. Mutation.

At this stage, small changes are introduced in the descendants generated by the crossing, with the aim of maintaining the population genetic variability. Each genetic code bit of sons and daughters has a 5% probability of being modified. To do this, random numbers are generated for each bit, and if the value is less than the mutation rate of 5%, the bit is changed. The mutated descendants, sons and daughters, are then combined into a new matrix, which represents the updated population. This population is taken again to the fitness evaluate stage, closing a cycle that will be repeated until the stopping criterion based on the target error is reached or the generation limit is simulated.

At the end of the routine, the genetic algorithm returns the fittest individual, with the smallest error found over the generations. This individual is then decoded to obtain the Rfit and Cfit values.

As a GA performance indicator, a graph is constructed that superimposes the simulated voltage curve (Eq (3)), obtained with Rfit and Cfit values of the best individual, on the curve collected from the CCCD test. To evaluate the accuracy of the definitive model, the average percentage error between the simulated voltage values of the fittest individual and the reference values (CCCD test) is calculated, as described in Eq (7), where N represents the total number of points of the analyzed data.


$$\text{Erro}\%=\frac{1}{N}\sum_{i=1}^{N}\left|\frac{V_{SC_{Best}individual}(i)-V_{SC\_target}(i)}{V_{SC\_target}(i)}\right|\times 100$$ (7)

### 2.5. Determination of Resistence (Rsd) and Supercapacitor Model Evaluation

Once Rfit and Cfit values are obtained via GA, a simulation is then carried out in PSIM to obtain the voltage curve for a complete cycle (charging, self-discharge and discharging), considering at this point the parameters obtained by GA and, then, this curve is compared with the reference curve, obtained by the CCCD test. With this, an adjustment is made for the resistance Rsd, comparing the self-discharge phase of the two curves. In addition, a definitive error of the SC model is determined, comparing the complete cycle of the two curves.

## 3. Results

**Fig 9:**
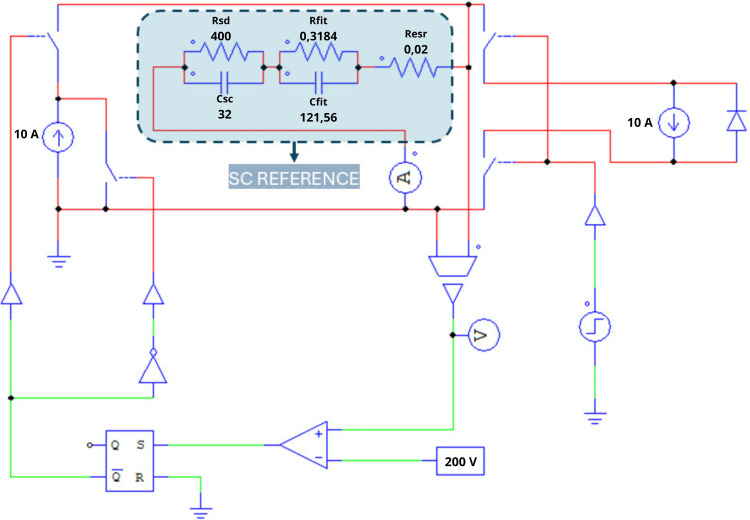
Circuit for a complete SC cycle (SC reference).

**Fig 10:**
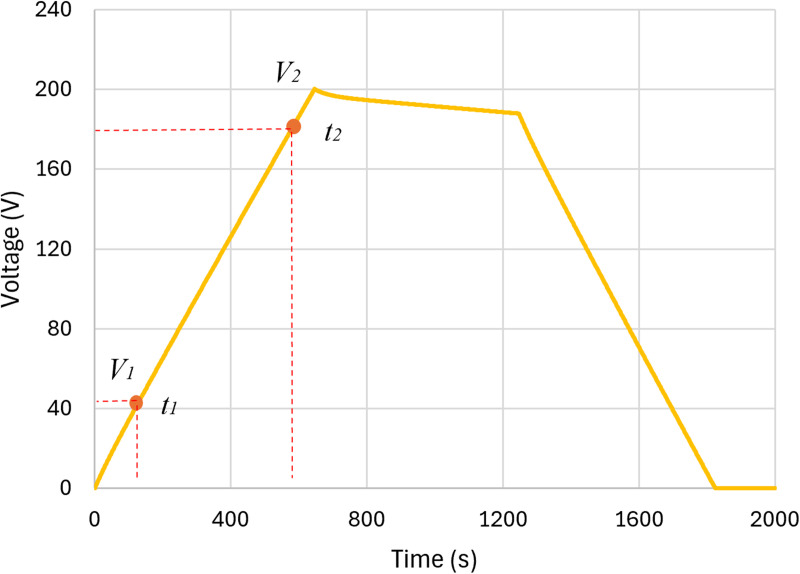
Charging curve of the target SC: Complete charging cycle, with key points for calculating Csc.

**Fig 11:**
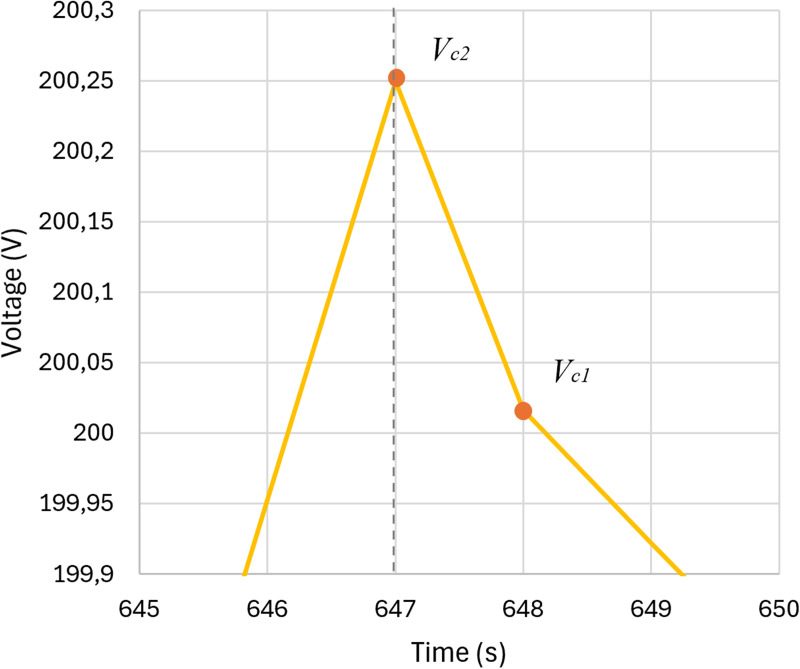
Charging curve of the target SC: Immediate voltage drop at the transition from charging to self-discharge, with key points for calculating Resr.

Using PSIM, the CCCD test was simulated using the circuit shown in Fig 9, considering the parameters of the supercapacitor to be modeled (Fig 2 and table 1). The resulting behavior is presented in the graph in Figs 10 and 11, together with the key points detailed in table 4. The two points in Fig 10 will be used for Resr calculations (Eq (1)), while the voltage values in Fig 11 (Vc1 and Vc2) and the charging current, will be used to determine Csc (Eq (2)).

**Table 4:** Points of interest for extracting the initial parameters, located in Fig 9.

*V1*	*t1*	*V2*	*t2*	*Vc1*	*Vc2*	*Ic*
40.25 V	120 s	180.29 V	580 s	200.016 V	200.25 V	10 A

For the results obtained in Figs 10 and 11, the following values of series equivalent resistance and capacitance were calculated: Resr equal to 0.023 Ω and Csc equal to 32.85 F. These values are inserted into Eq (3) and then GA is used to determine the resistance (Rfit) and capacitance (Cfit) values.

Table 5 shows the results regarding the GA performance, where the number of generations and individuals created until the stop can be seen, with the final error around 0.74%. Although the target error of 0.5% has not been achieved, the error obtained is small enough for supercapacitor modeling purposes.

**Table 5:** Genetic Algorithm Performance.

%Error_Target	%Error obtained	Generations	Generated solutions (individuals)
0.5%	0.74%	100,000	100,000,000

**Fig 12:**
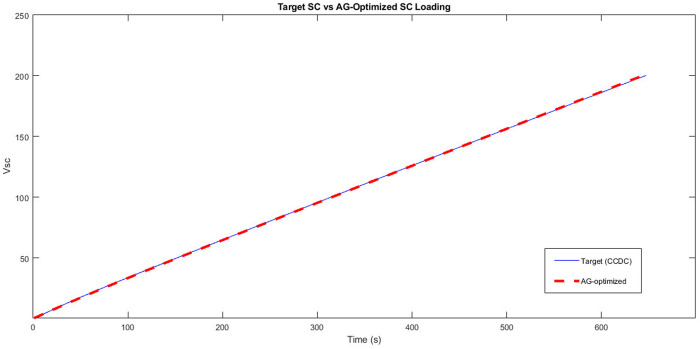
Charging curve modeled by the settings determined by the GA compared with the target charging curve.

In Fig 12, a comparison is presented between the charging voltage of the supercapacitor to be modeled (taken from the CCCD test) and the charging voltage of the fittest individual (best solution found) found by GA (obtained by Eq (3)). As can be seen in Fig 12, the two curves are very close, showing the GA efficiency in determining Rfit and Cfit.

To complement and confirm the supercapacitor model, a circuit is assembled with the determined values, where Rsd is adjusted until the best approximation to the self-discharge observed in the initial curve is obtained (CCCD test).

**Fig 13:**
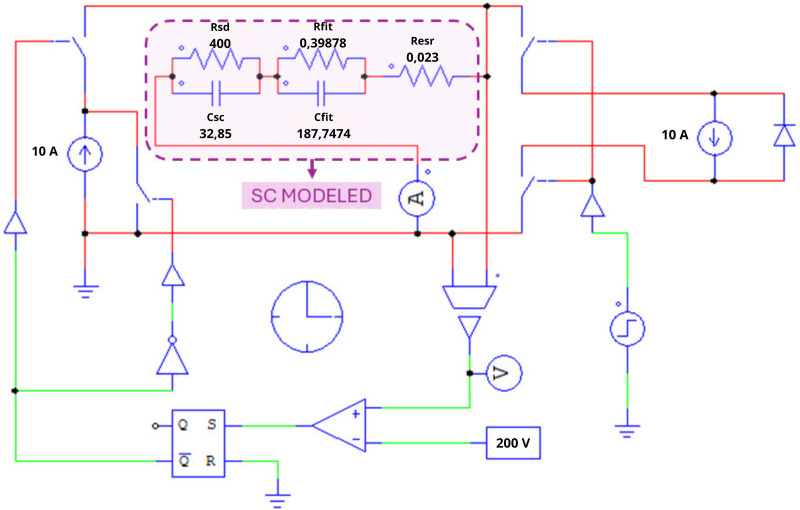
Circuit for a complete SC cycle (SC modeled).

All parameters obtained, both by the CCCD test analysis and those optimized by GA, are presented in table 6. The charge and discharge circuit for such values is shown in Fig 13.

**Table 6:** Key parameters of the modeled supercapacitor.

C_SC_	R_ESR_	C_fit_	R_fit_	R_sd_
32.85 F	0.023 Ω	187.7474 F	0.39878 Ω	400 Ω

**Fig 14:**
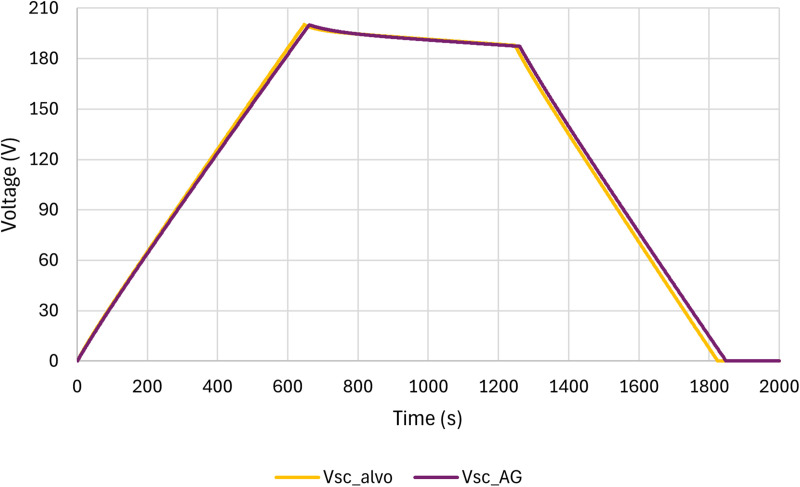
Comparison between the target voltage curve and the modeled voltage curve with GA.

The comparison between the target voltage curve (CCCD test – Circuit in Fig 9) and the modeled voltage curve with GA (Circuit in Fig 13), for a complete charge and discharge cycle, is shown in Fig 14. As can be seen in Fig 14, the method using GA obtained a good result, since the behaviors of the two curves are very similar in all stages, highlighting a greater difference during discharging. The percentage errors at each step are shown in table 7.

**Table 7:** Accuracy of the supercapacitor modelling.

General_%Error	% Error during charge	%Error during self-discharge	%Error during discharge
3.93%	2.20%	0.23%	10.07%

## 4. Discussion

Charge and discharge tests performed at PSIM demonstrated the effectiveness of the genetic algorithm in optimizing the dynamic parameters Rfit and Cfit of the supercapacitor, with a strong correlation between the voltage curves of the target supercapacitor and the digital model. The average percentage error between the reference and model data indicated high accuracy of the model, despite small discrepancies during discharge. Compared to previous studies, this work stands out for its fine-tuning and detailed analysis of the percentage error. The proposed methodology can be applied to other energy storage devices, with future research focusing on the integration of advanced sensors and multi-objective optimization algorithms for modeling a physical SC.

The electrical supercapacitor model proposed in this article cannot be applied to the other types of supercapacitors, namely: pseudocapacitors and hybrid supercapacitors is inadequate to these types of supercapacitors because of:

i Differences on energy storage mechanisms, redox reactions (faradaic processes) on pseudocapacitors and electrochemical volumetric processes on hybrid supercapacitor.
ii Complexity on dynamical behaviour of pseudocapacitor and hybrid supercapacitors that exhibit nonlinear responses (e.g., capacitance dependence on voltage or charge/discharge rate). The proposed model does not capture such nuances.
iii Failure to represent multiple time scales, hybrid supercapacitors and pseudocapacitors a have multiple time constants due the coexistence of fast processes (electrostatic) and slow process (electrochemical). The proposed model uses a single RC branch for the dynamics.

Despite all these limitations, the model is effective to represent EDLCs due to the following reasons:

i Alignment with the electrostatic storage mechanism: EDLCs operate by purely electrostatic storage (electrical double layer), without chemical reactions. The simplified model, composed of resistances (series and self-discharge) and fixed capacitances, directly reflects this physical behaviour, ignoring unnecessary complexity such as the dependency on voltage or Faradaic processes.
ii Simplicity and practicality: The circuit has only five parameters (RESR, RSD, CSC), identified by simple experimental tests (charge/discharge with constant current and self-discharge) and adjusted by the Genetic Algorithm application (RFIT and CFIT). Such simplicity allows easy implementation in tools such as PSpice, keeping the accuracy for typical applications of EDLCs.
iii Computational efficiency: This application is part of a larger project involving the use of Digital Twin technology for a hybrid stand-alone PV system in which the supercapacitors are of electrical double layer type. The simplified architecture minimizes the computational overhead in simulations, making it particularly suitable for digital twin applications.

## 5. Conclusions

In this study, an equivalent electrical model of a supercapacitor using a genetic algorithm (GA) to optimize dynamic parameters was developed and validated. The adopted approach integrated theoretical and experimental methods, resulting in a precise and robust digital model capable of replicating the supercapacitor’s physical behavior with high fidelity.

Analysis of the results demonstrated that the GA-optimized model exhibited an overall percentage error of only 3.93%, with particular precision in charging (2.20%) and self-discharge (0.23%). These values indicate an excellent correlation between experimental and simulated data, validating the effectiveness of the proposed model. However, a larger error was observed in discharging (10.07%), suggesting that future research should focus on improving modeling for this specific stage. Additionally, the detailed experimental validation reinforces the need to combine theoretical and practical approaches to develop models that are not only accurate but also applicable in real-world scenarios. The integration of such methodologies is crucial for the effective implementation of Digital Twins, which can revolutionize the management and optimization of energy storage systems. Therefore, this work not only contributes to advancing supercapacitor modeling techniques but also paves the way for the application of Digital Twins in energy storage systems. The precision and efficiency achieved by the developed model highlight the importance of continuing to explore and improve these technologies, aiming for significant improvements in the performance and energy efficiency of industrial applications and renewable energy generation.

## Supporting information

S1 File Input Data to GA. (XLSX)

S2 File Comparison of Target SC and Optimized SC. (XLSX)
